# Supplementary material for: Andreev molecules in semiconductor nanowire double quantum dots
Source: Nat Commun. 2017 Sep 19;8:585. doi: 10.1038/s41467-017-00665-7 (PMC5605684; doi:10.1038/s41467-017-00665-7)
Supplement: Supplementary file 2 — Supplementary Information [file 41467_2017_665_MOESM2_ESM.pdf]

SI GUIDE

File Name: Supplementary Information
Description: Supplementary Figures, Supplementary Notes and Supplementary References.

File Name: Peer Review File
Description:

SUPPLEMENTARY FIGURES

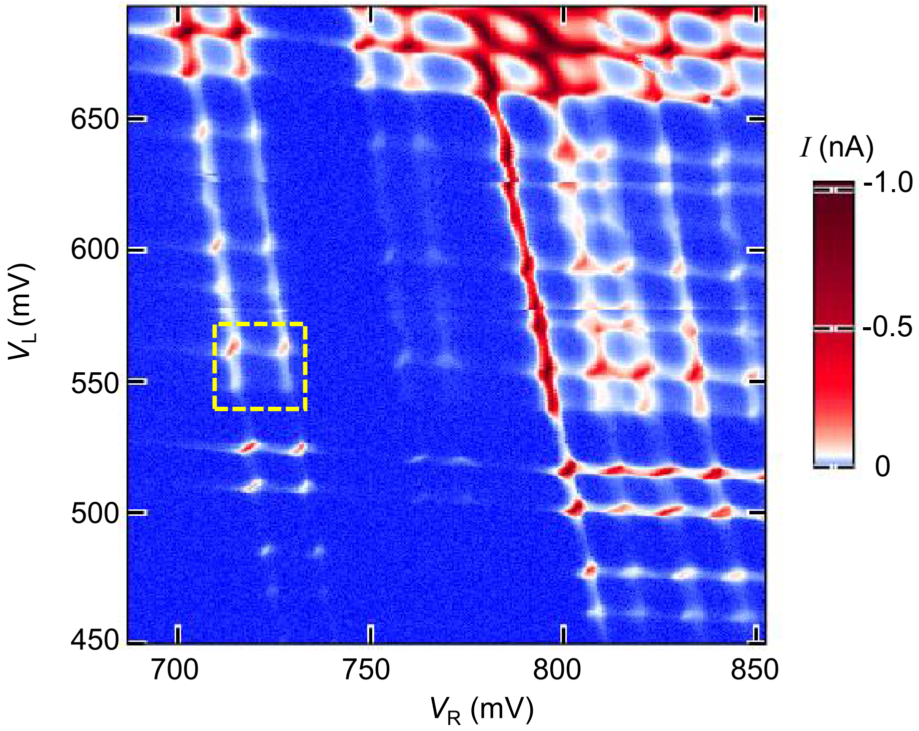

Supplementary Figure 1. **Expanded double dot stability diagram.** Double dot stability diagram at the bias of -200 μV and at zero magnetic field in a large gate voltage range. Measurements in the main text focus on the window enclosed by the yellow square. We note that the left dot has a charge instability, meaning that the features shift along the vertical axis spontaneously. The instability, however, is tractable and we are able to tune V_{L} to compensate the voltage change when it occurs. This results in mismatch in V_{L} ranges in conductance maps taken at different times.

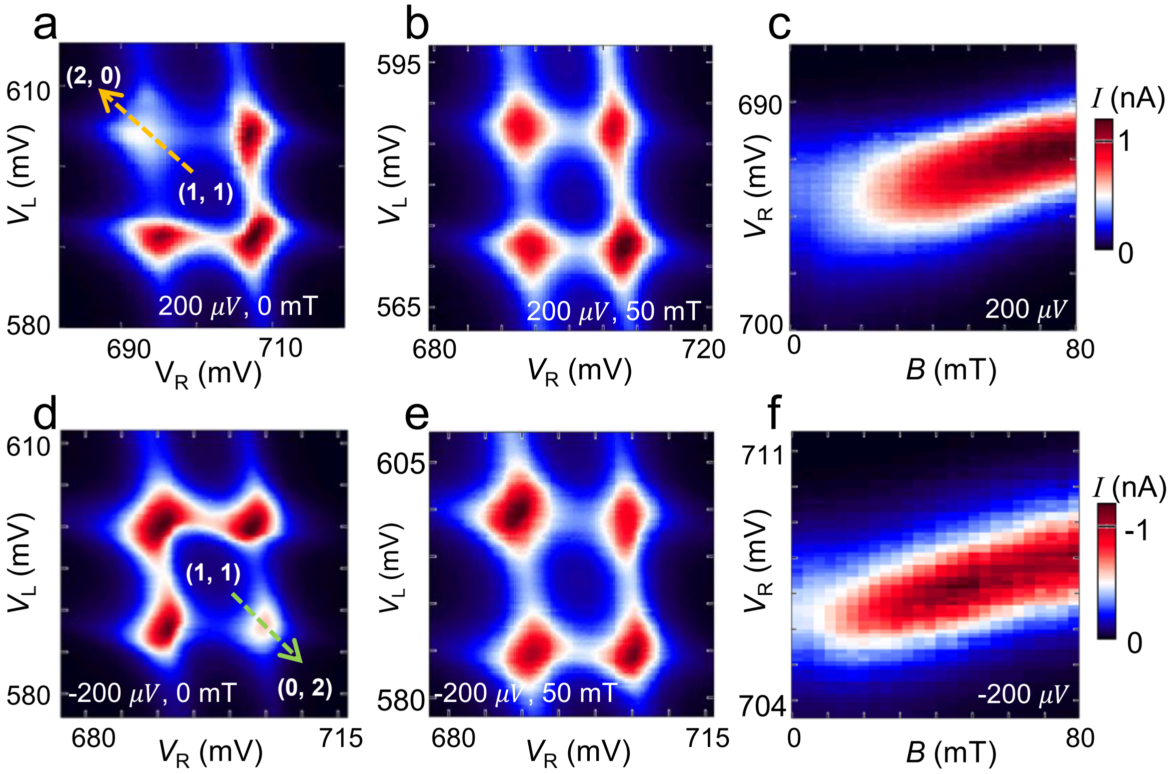

Supplementary Figure 2. **Spin Blockade.** **a**, At positive bias of 200 μV the (1, 1)↔(2,0) degeneracy point has smaller current. **b**, At the reverse bias the (1, 1)↔(0,2) degeneracy point has the smaller current. **c, d**, Spin blockade is lifted by a magnetic field of 50 mT, with all four degeneracy points showing similar current levels. In **e, f**, gates are swept through the spin blockade region (arrows in **a** and in **b**) as magnetic field is stepped. The current has a zero-field dip, consistent with spin blockade reported previously for InSb double quantum dots[1].

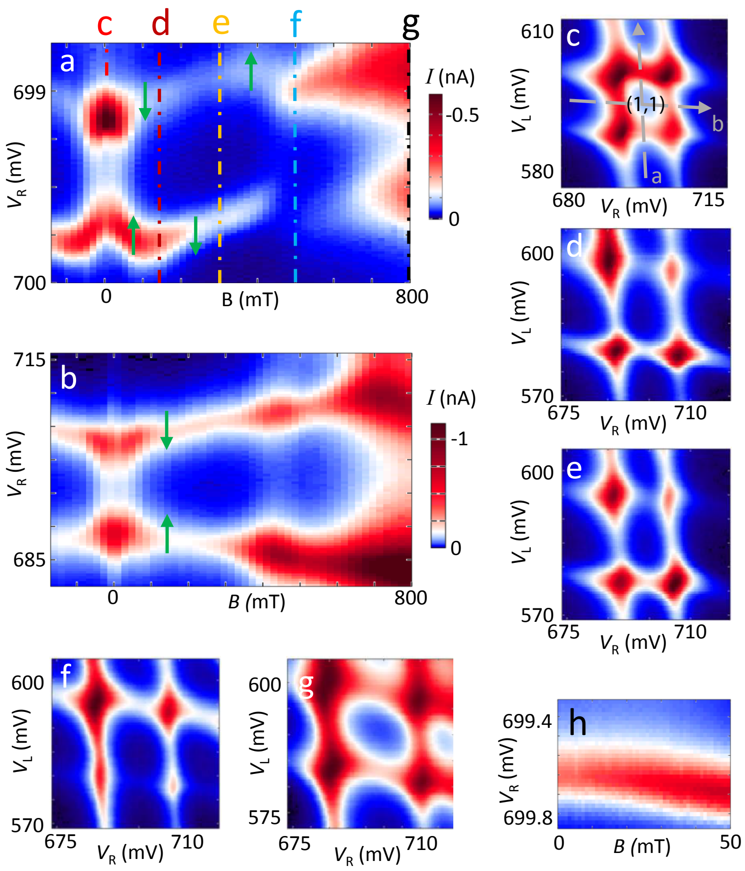

Supplementary Figure 3. **Magnetic field evolution of the charge stability diagram. a-b,** The evolution of current along the line cuts a and b shown in panel **c**, V_{bias} = -200 μV. For B $\leq$ 100 mT, the high current lines that define the (1,1) region move apart. At higher fields, the lines exhibit kinks. They are are consistent with previously reported ground state spin transitions in InSb nanowire quantum dots[2]. Spins of the electrons added to the dot are depicted by arrows along the high current lines. **c-g,** The double dot stability diagrams at 0, 140, 300, 500, 800 mT, V_{bias} = -200 μV. We notice that the higher current lines connected the spin blockade points (lower branch of **a** and higher branch of **b**) also undergo lower current at zero field. It is specially demonstrated with a zoomed-in scan of the lower branch from 0 to 50 mT (**h**). The color bar of **b** is on its right side. The other plots share the color bar on the right side of **a**.

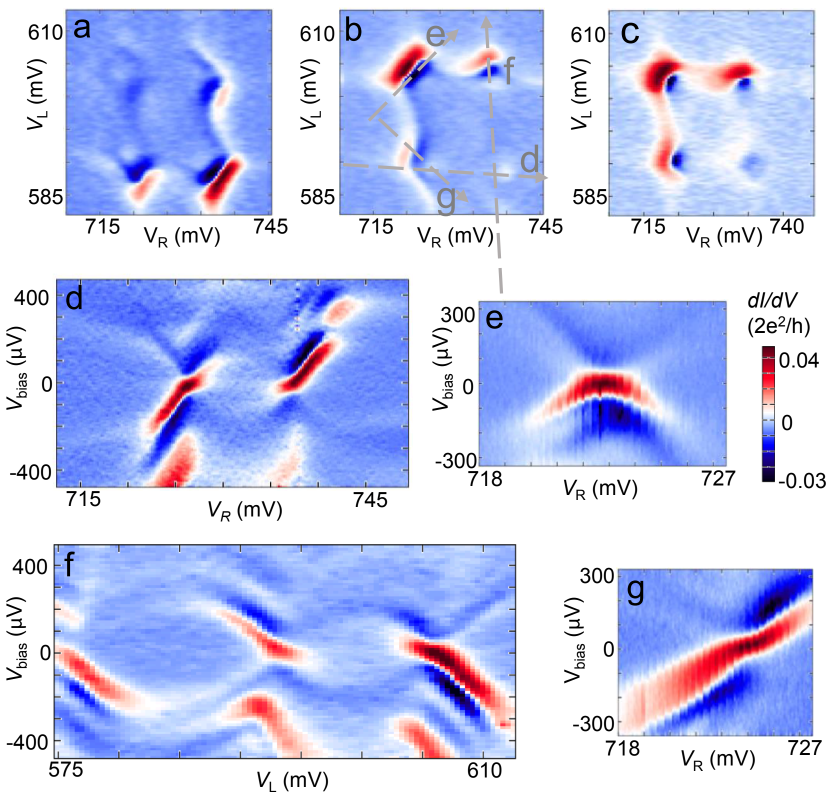

Supplementary Figure 4. **Complimentary data on Andreev Molecular States.** Spectroscopy measurements of Andreev molecular resonances complimentary to Fig.2 and Fig.3 of the main text. **a-c**, Gate vs. gate differential conductance diagrams at the bias of (a) 200 μV, (b) -200 μV and (c) -100 μV. **d-g**, Source-drain bias spectroscopy scans along various cuts depicted by the arrows in **b**. **d**, With the left dot fixed at its degeneracy point. **f**, With the right dot fixed at its degeneracy point. Here we extend the plot to display adjacent Andreev loops. **e**, Along the symmetric cut through the (1,0)$\leftrightarrow$(2,1) degeneracy point. **g**, Along the detuning cut through the (1,0)$\leftrightarrow$(0,1) degeneracy point.

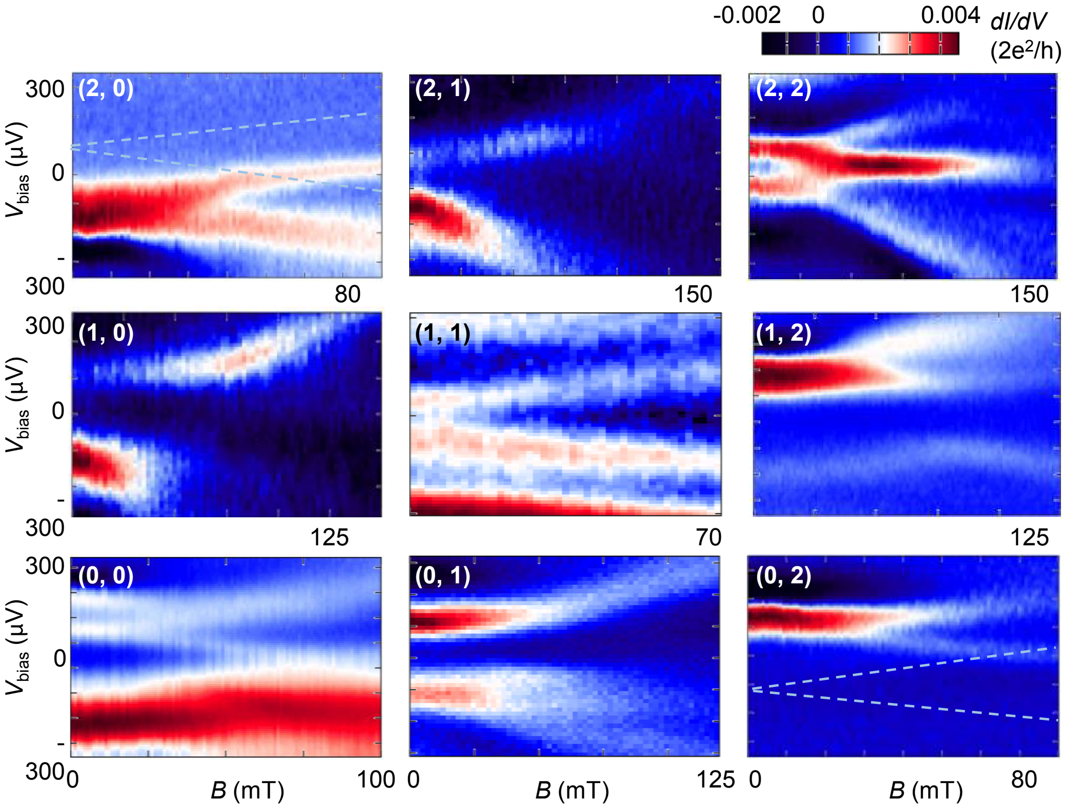

Supplementary Figure 5. **Spin map.** The magnetic field dependence of the subgap resonances in the 3x3 double dot configurations around (1,1). Within each configuration, the magnetic field dependence of the resonances is measured at a few fixed gate voltages. Scans in each configuration demonstrate consistent magnetic field behavior and one typical scan is presented here. Note that the positive (negative) bias branch in (2,0) ((0,2)) is missing, which is a consequence of spin blockade and asymmetric couplings.

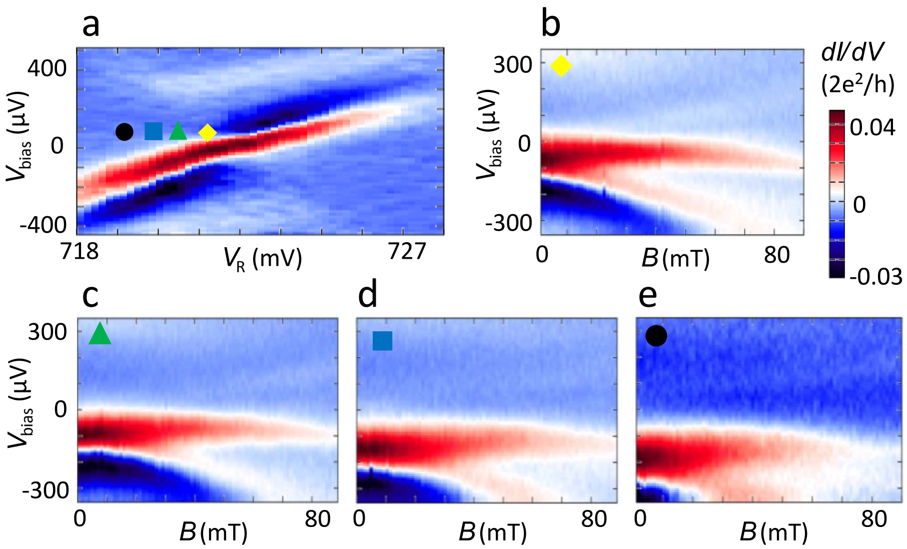

Supplementary Figure 6. **Detailed magnetic field data in the (2,1) configuration.** The magnetic field dependence in (2,1) at various spots away from the degeneracy point. **a**, The spectroscopy along the detuning cut through the (2,1)↔(1,2) degeneracy point. **b-e**, A series of bias vs. field scans in (2,1) at spots farther and farther away from the degeneracy point, depicted by the yellow diamond, green triangle, blue square and black dot marked in panel **a**. This shows that qualitatively the same features are observed within the same quadrant of the double dot stability diagram in Supplementary Figure 5.

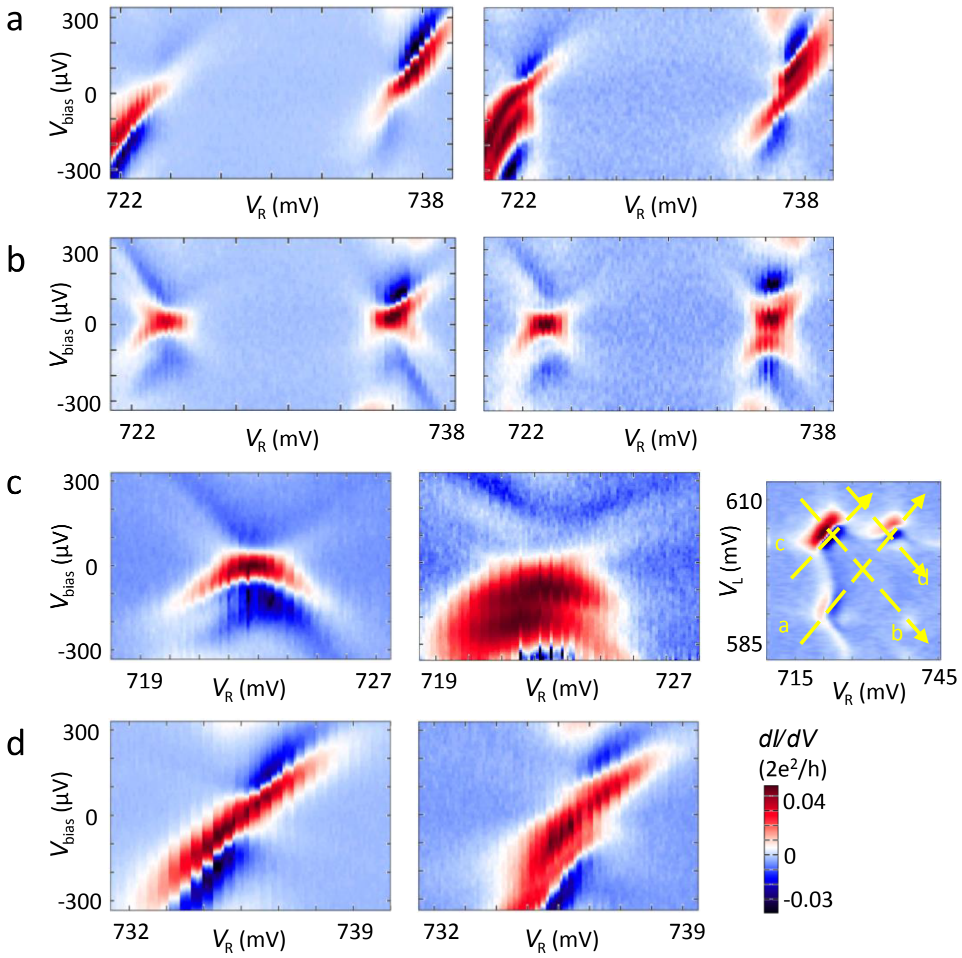

Supplementary Figure 7. **The source-drain bias spectroscopy at zero and finite fields. a-d,** The spectroscopy measurements at 0 mT (left) and 35 mT (right) along different cuts depicted by the yellow arrows in **e** which is the same as Supplementary Figure 4**b**. **a-b**, For both bias directions, resonances are doubled at finite field in the (even, even) configurations; while in (1,1) a resonance does not split. **c**, Along the energy shift cut through the (2,0)↔(1,1) degeneracy point. **d**, Along the detuning cuts through (2,1)↔(1,2) degeneracy point.

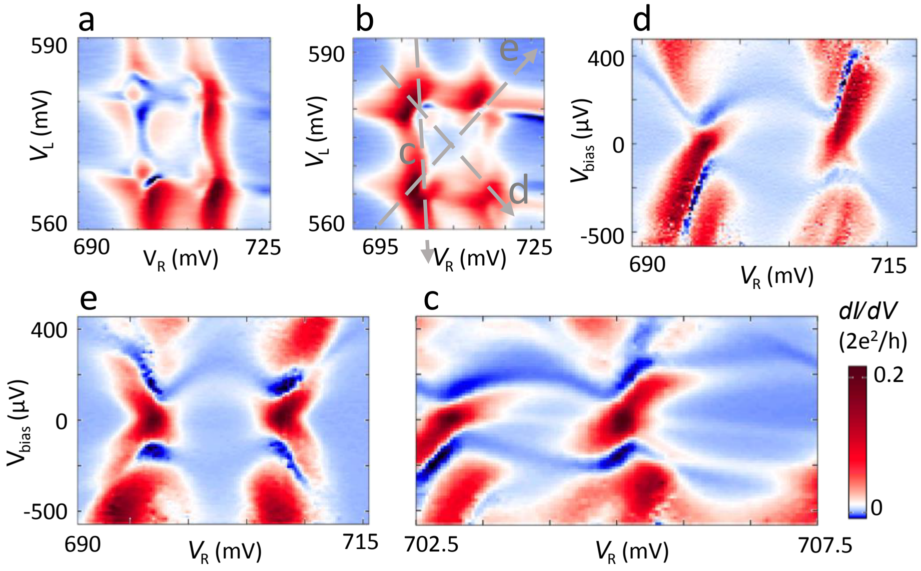

Supplementary Figure 8. **Strong interdot coupling regime.** Data in this plot and Supplementary Figure 9 are obtained after the device has been re-tuned to lower the interdot barrier by adjusting gate V_t from -685 mV to -635 mV, and all other gates to keep the same dot occupations. $V_{\Gamma L}$ and $V_{\Gamma R}$ remain almost the same. **a-b**, The gate vs. gate differential conductance diagrams at biases of 200 μV and -200 μV. **c** and **d**, The spectroscopy scans along the detuning and energy shift cuts. **e**, scan the left dot when the right dot is fixed at the degeneracy point. The spectra are qualitatively similar to those in the weaker interdot coupling regime. However, owing to the stronger interdot coupling, a few features which are less clearly resolved in the weaker interdot coupling regime are more pronounced here. First, the resonances away from the degeneracy points are clearly visible. Second, the close-to-zero-bias resonances, that appear within the loop-like resonances are resolved. These resonances appear due to proximity to charge degeneracy in the right dot, they are further discussed in Supplementary Figure 12.

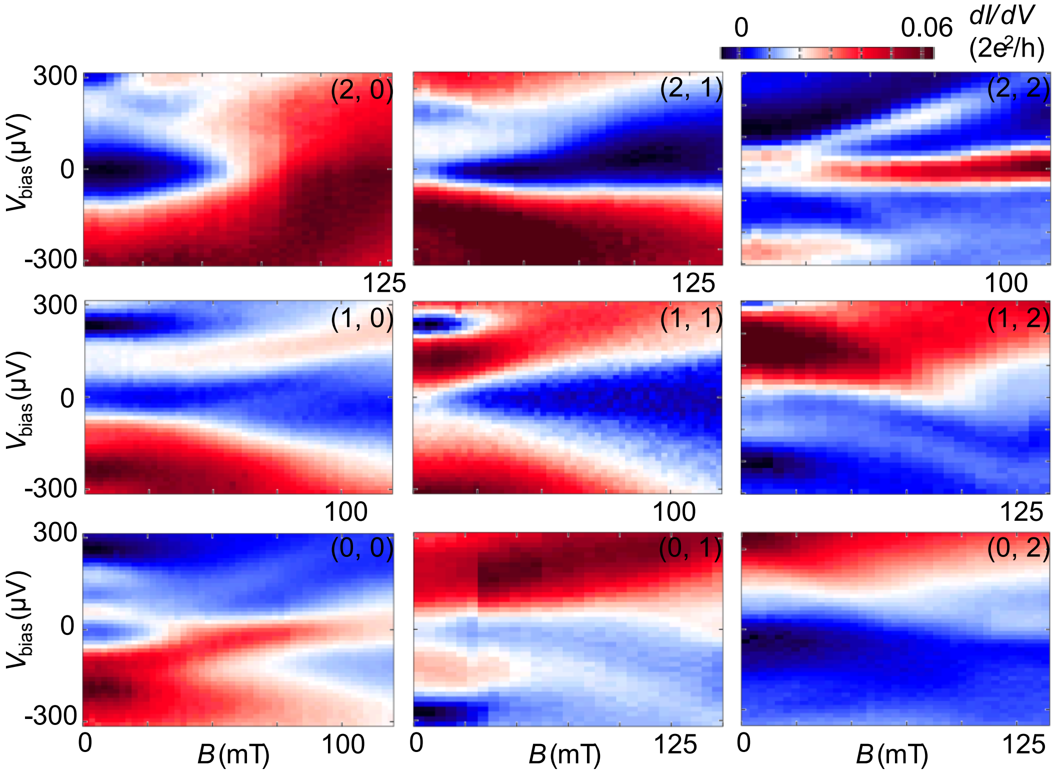

Supplementary Figure 9. **The spin map in the stronger interdot coupling regime.** Spin map in the stronger interdot coupling regime ($V_t = -635$ mV). Magnetrospectroscopy is performed in the 3x3 double dot configurations around (1,1) in the stronger interdot coupling regime. In each configuration a typical example is presented. Although the resonances are more broadened, qualitatively similar magnetic field dependencies are observed here as in Supplementary Figure 5. That is, resonances don't split and moves away from zero bias in (1,1), resonances split in the (even, even) configurations, and resonances exhibit kinks at positive (negative) bias and broadened structures at negative (positive) bias in (1,0) and (2,1) ((0,1) and (1,2)).

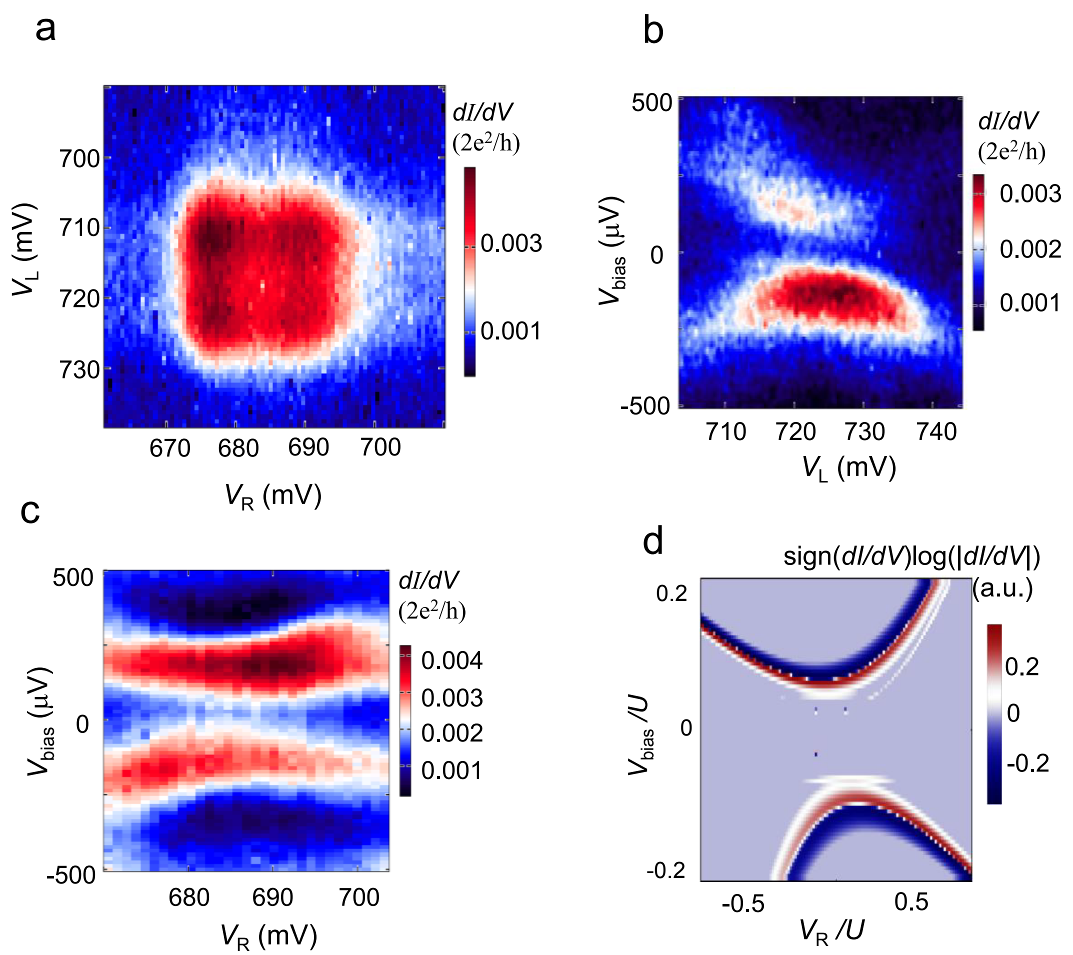

Supplementary Figure 10. **Strong superconductor-quantum dot coupling regime.** The spectroscopy of the double dot system in strong superconductor-quantum dot coupling regime. **a,** dI/dV as a function of V_{L} and an V_{R} with a fixed bias at -120 μV. In this strong coupling regime the four degeneracy points are broadened due to increased tunnel coupling to the leads. **b(c),** the resonances as a function of V_{L} (V_{R}) when V_{R} (V_{L}) is set to 687 mV (717 mV) respectively. In this regime, the single dot ground states are singlets and the resonances loops have anti-crossings. The resonances are less sharp because the quantum dots outgoing and incoming barriers are very open. The data are from another. **d,** the simulated plot in the strong coupling regime, when left dot chemical potential is fixed to 0 and right dot chemical potential is swept. The simulation also reproduces the anticrossing observed in experiment.

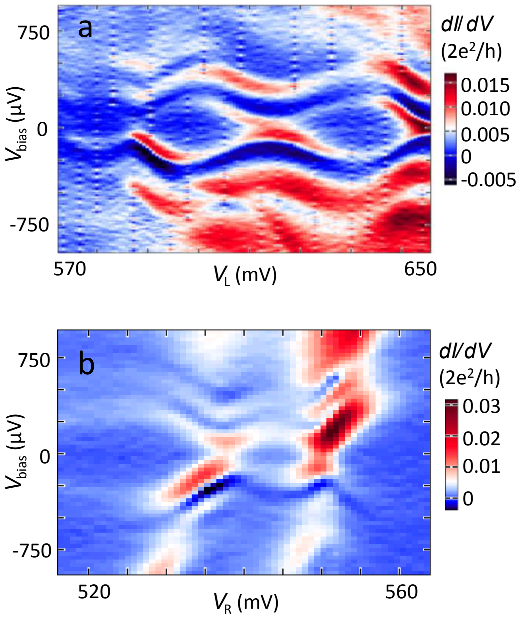

Supplementary Figure 11. **High bias scans revealing multiple loops of single dots.** In **a** we show the spectroscopy of the left single dot when the right dot is removed. The system is therefore a single quantum dot coupled to a superconducting lead and probed by the other superconducting lead that is weakly coupled to the dot. Besides the lowest bias loop-like resonances, we observe copies of these resonances at higher biases. They demonstrate non-linear gate dependence of the same pace of the lowest bias loops. Both lowest bias loops and higher bias resonances are followed by negative differential conductance peaks. These features are observed in the right single dot as well (**b**). We note that the copies are present only in systems with both leads superconducting. When an Andreev bound state is probed by a normal lead, only one loop is observed within the gap. The copies of the Andreev states may originate from quasiparticle peaks in the tunneling probes and multiple Andreev reflections. They are being addressed in a future publication. In this paper we focus on the resonances closest to zero bias.

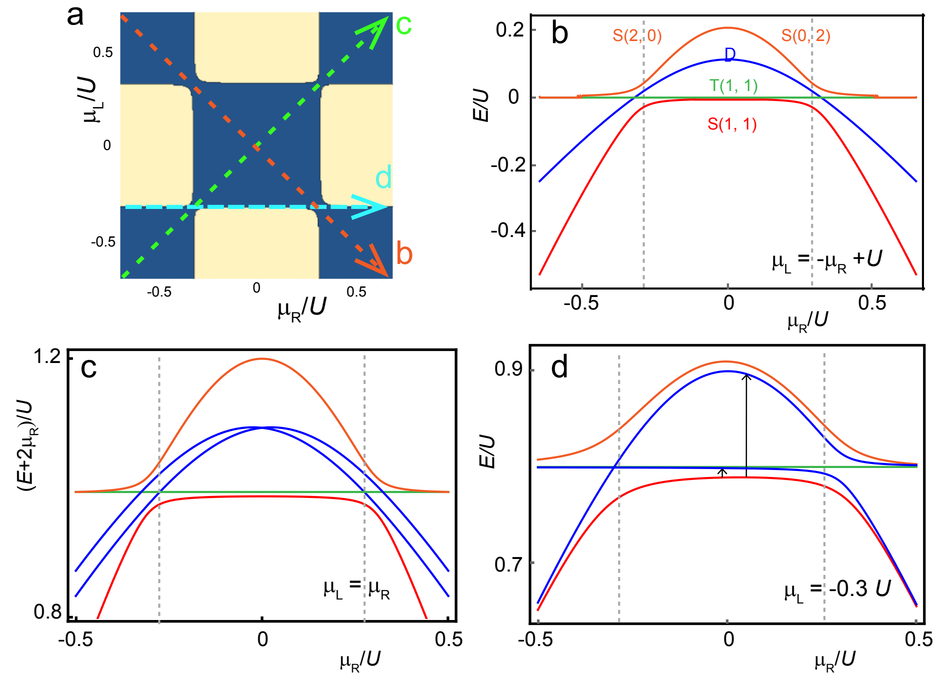

Supplementary Figure 12. **Simulated spectra of the S-QD-QD-S system.** Simulated spectra of the system at zero bias. Throughout the paper, the simulated spectra and transport plots use the same parameters, i.e., Andreev reflection matrix element $\tau_{\mathrm{L/R}}/U = 0.4$, inter-dot tunneling matrix element $t/U = 0.01$ in terms of the on-site interaction strength U. **a**, The simulated diagram showing the ground state parities as a function of left and right dot chemical potential (μ_L and μ_R). Blue regimes have total parities of even and yellow regimes have total parities of odd. **b-d**, Bias scans along the cuts depicted in **a**. These spectra explain the corresponding bias vs. gate subgap conductance measurements. We notice that, interestingly, in the measurements where one dot is fixed at the degeneracy point (Fig.3**a-b** in the main text and Supplementary Figure 4**d,f**), additional close-to-zero-bias resonances are observed inside the loop-like structure. They can be explained by **d** where besides the transition from the ground state to the higher doublet state (tall arrow), there exist the transition to the lower doublet state which requires little energy (short arrow). They are associated with adding or removing an electron in the degenerate dot and therefore they cost little energy.

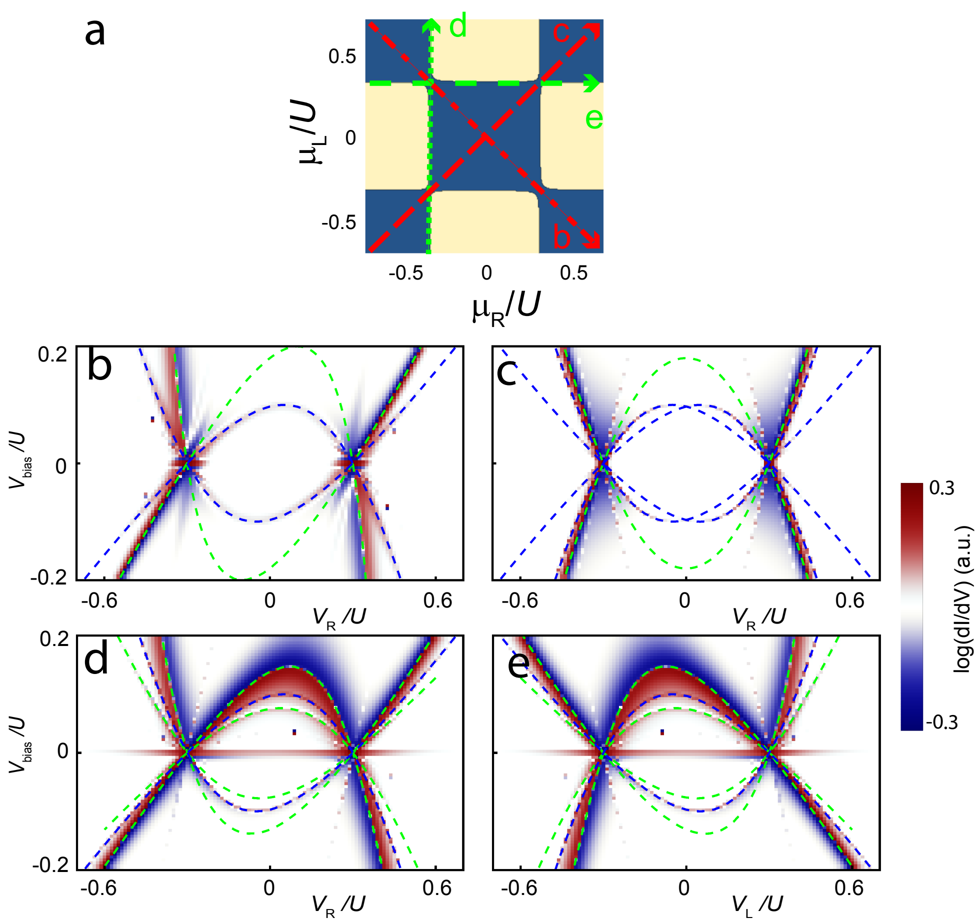

Supplementary Figure 13. Simulated transport plots. The diagram (**a**) used to illustrate the line cuts for **b-e** is the same as Supplementary Figure 12**a**. **b-e** are bias vs. gate plots along cuts depicted in **a**. The green dashed lines indicate the bias voltage at which the levels on the quantum dots come into resonance and the blue dashed lines indicate when the bias voltage exceeds the Andreev level spacing. Notice that additional resonances near zero bias are shown inside the loop-like resonances (**d, e**), which is consistent with the experimental observations.

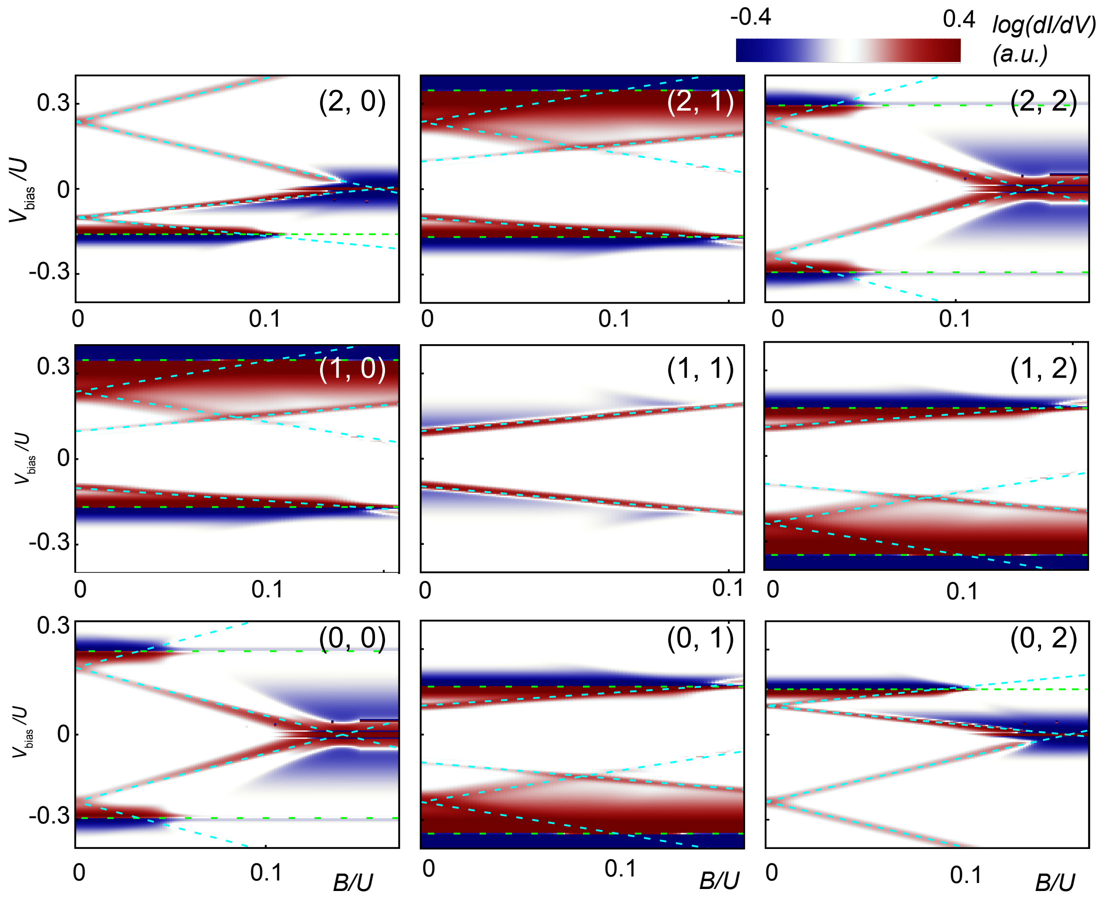

Supplementary Figure 14. **The simulated spin map.** Simulated magnetic field dependence of the 3x3 configurations around (1,1). Besides the good fitting between measurements and simulations in each individual configuration, the experimental (Supplementary Figure 5) and the simulated maps exhibit impressively similar patterns. First, (0,0) and (2,2) show resonances symmetrically at both biases, while only the negative (positive) branch has high conductance in (2,0) ((0,2)). Second, in (1,0) and (2,1) the kinks occur at the positive bias while in (0,1) and (1,2) the kinks occur at the negative bias. We notice that even with stronger interdot coupling (Supplementary Figure 9), such consistency is still valid.

SUPPLEMENTARY NOTE 1 ANDREEV MOLECULES AT ZERO BIAS

In this section we explicitly write down the model Hamiltonians for Andreev atoms and molecules that were used to produce Fig. 1b,c of the main text.

The minimal model for Andreev bound states (Andreev atomic states) in a superconductor-quantum-dot (S-QD) system at zero bias consists of a single level quantum dot coupled to the superconducting reservoir. The model is described by the Hamiltonian

$$H_{\text{S-QD}} = \sum_{\sigma=\{\uparrow,\downarrow\}} \varepsilon_\sigma n_\sigma + U n_\uparrow n_\downarrow - \left(\tau d_\uparrow^\dagger d_\downarrow^\dagger + \text{H.c.}\right), \tag{1}$$

where ε_σ is the energy of an electron with spin σ to be on the quantum dot which is set by the gate voltage and the magnetic field, $n_\sigma = d_\sigma^\dagger d_\sigma$ is the number operator for electrons with spin σ on the dot, U is the charging energy for double occupancy of the dot, τ describes the strength of Andreev reflection of electrons from the superconductor and $d_\sigma^\dagger$ and d_σ are the electron creation and annihilation operators. The spectrum of this Hamiltonian consists of two spin-singlets ($\sin(\theta)|0\rangle + \cos(\theta)|\uparrow\downarrow\rangle$ and $\cos(\theta)|0\rangle - \sin(\theta)|\uparrow\downarrow\rangle$) and a spin-doublet ($|\uparrow\rangle$ and $|\downarrow\rangle$). The parity of the ground state, as well as the angle θ, is determined by the coupling constants and can be tuned from even to odd to even by varying $\varepsilon_\uparrow$ and $\varepsilon_\downarrow$. The lower of the spin singlet states and the spin doublet states are plotted in Fig. 1b.

To describe Andreev molecular states at zero bias we need to couple two Andreev atoms. The corresponding Hamiltonian becomes

$$H_{\text{S-QD-QD-S}} = \sum_{i=\{L,R\}} \left[\sum_{\sigma=\{\uparrow,\downarrow\}} \varepsilon_{i,\sigma} n_{i,\sigma} + U_i n_{i,\uparrow} n_{i,\downarrow} - \left(\tau_i d_{i,\uparrow}^\dagger d_{i,\downarrow}^\dagger + \text{H.c.}\right) \right] \tag{2}$$

$$- t \sum_{\sigma=\{\uparrow,\downarrow\}} \left(d_{L,\sigma}^\dagger d_{R,\sigma} + \text{H.c.} \right), \tag{3}$$

where the subscripts L and R stand for the left and the right quantum dots and t is the inter-dot tunneling matrix element. The eigenstates of $H_{\text{S-QD-QD-S}}$ are the Andreev molecular states plotted in Fig. 1c.

In the following section, we discuss a more detailed model of Andreev molecular states at nonzero bias voltages, which describes both Andreev reflections and inter-dot coupling while keeping track of the charging energy of the two superconducting leads.

SUPPLEMENTARY NOTE 2 DETAILED DESCRIPTION OF OUR TRANSPORT MODEL

Model Hamiltonian

We use two main ingredients to setup our detailed model of the transport properties of the superconductor-double-dot-superconductor (S-QD-QD-S) device. The first ingredient is a ladder of Andreev molecular states of the S-QD-QD-S system. These states are a result of the interplay of Andreev reflections and hybridization between the quantum dots. The second ingredient is a low, but finite, density of sub-gap quasi-particle states in the leads. The resonant tunneling of these sub-gap quasi-particles into the double dot subsystem is responsible, within our model, for driving transitions between the eigenstates of the S-QD-QD-S system and hence for the experimentally observed sub-gap transport features. To account

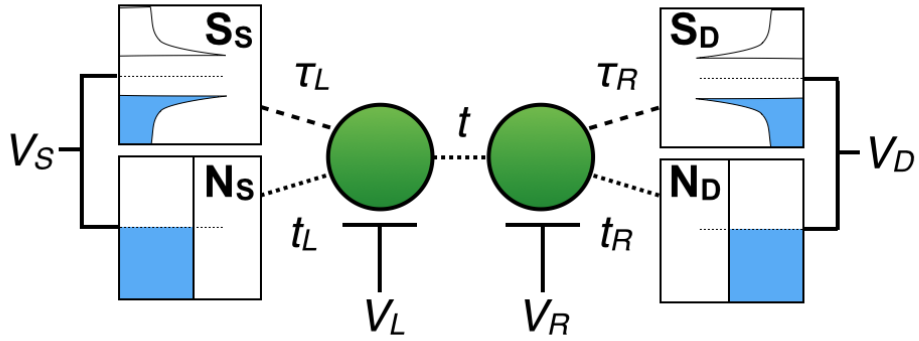

Supplementary Figure 15. Hybrid superconductor-double-dot-superconductor system, consisting of an array of two quantum dots tunnel-coupled to superconducting leads. Each lead is modeled as having a standard BCS superconducting (**S**) component and a normal metal (**N**) component. The coupling to the BCS components give rise to Andreev reflection processes, whereas the coupling to the normal components provide low-energy electronic excitations which are responsible for sub-gap transport. The strength of the inter-dot tunneling is set by t, while τ_L and τ_R (t_L and t_R) control the coupling of the left and right dots to the superconducting (normal) components of the left and right leads. The leads are biased by the source (drain) voltages $V_{S(D)}$ and the chemical potential on the left (right) dot is controlled by the side-gate voltage $V_{L(R)}$.

for the presence of sub-gap quasi-particles, we model each lead as having a superconducting component and a normal metal component [see Fig. 15]. The superconducting component

is a conventional BCS superconductor with a hard gap Δ, which provides a condensate of Cooper pairs and drives Andreev reflection processes. The normal component, which we model as a non-interacting Fermi gas with a low density of states at the Fermi surface, provides the low energy electronic excitations that are necessary for sub-gap transport. Finally, to model the application of bias, V_{bias}, we tie the electro-chemical potentials of the two components together and fix them to the applied bias voltage [see Fig. 15].

One of the key features observed in the experimental data are discrete and narrow Andreev bound state-like features. As the strong resonant tunneling of electrons from the normal component of the leads to the quantum dot sub-system tends to broaden the discrete levels of the quantum dot sub-system, we restrict our modeling to the regime where single-electron tunneling (between the quantum dot system and the normal metal component of the leads) is the weakest coupling in the system.

Our transport model is encoded by the Hamiltonian

$$H = H_{\mathrm{QD}} + H_{\mathrm{S}} + H_{T,S} + H_N + H_{T,N}, \tag{4}$$

where H_{QD} describes the double-dot subsystem, H_{S} describes the electro-chemical potential energy of the Cooper pairs in the superconducting leads, $H_{T,S}$ describes Andreev reflection, H_N is the Hamiltonian of the normal component of the leads, and $H_{T,N}$ describes the tunneling between the QDs and the normal components of the leads. H_{QD} is given by

$$H_{\mathrm{QD}} = \sum_{j\sigma} \varepsilon_{j\sigma} n_{j\sigma} + U \sum_{j} n_{j\uparrow} n_{j\downarrow} - t \sum_{\sigma} \left(d^{\dagger}_{R,\sigma} d_{L\sigma} + d^{\dagger}_{L\sigma} d_{R,\sigma} \right), \tag{5}$$

where $n_{j\sigma} = d^{\dagger}_{j\sigma} d_{j\sigma}$ is the number operator of the electrons on QD $j = \{L, R\}$ with spin σ, energy $\varepsilon_{j\sigma}$ (controlled by the electro-chemical potential in quantum dot j). The strength of the Coulomb repulsion and of the inter-dot coupling is set by U and t, respectively. The model Hamiltonian for the leads is a combination of the superconducting component

$$H_{\mathrm{S}} = \sum_{j \in \{S,D\}} eV_j N_j, \tag{6}$$

and the normal component

$$H_N = \sum_{j \in \{S,D\}} \sum_{\mathbf{k}\sigma} \left(\xi_{\mathbf{k}} + eV_j \right) c^{\dagger}_{j\mathbf{k}\sigma} c_{j\mathbf{k}\sigma}, \tag{7}$$

where $j = \{S, D\}$ indicates the source and drain leads, N_j represents the electron number operator for the superconducting component, $c^{\dagger}_{j\mathbf{k}\sigma}$ ($c_{j\mathbf{k}\sigma}$) creates (annihilates) an electron

with momentum $\mathbf{k}$ and spin σ with energy $\xi_{\mathbf{k}}$ in the normal component of lead j, and both components are biased by the same voltages V_j. The Andreev reflection (i.e. pair tunneling) is described by the Hamiltonian

$$H_{T,S} = -\tau_L S_S^+ d_{L\downarrow} d_{L\uparrow} - \tau_R S_D^+ d_{R\downarrow} d_{R\uparrow} + \text{H.c.}, \tag{8}$$

where the operator S_j^+ increases the number of electrons in the superconducting condensate of the j-th superconducting lead by two: $N_j \to N_j + 2$. Keeping track of the number of electrons in the superconducting condensates in the two leads is an essential feature of the model that allows us to describe Andreev reflection between the QDs and both leads when there is a voltage difference between the leads[3]. The coupling between the QDs and the normal leads is given by the conventional tunneling Hamiltonian

$$H_{T,N} = -t_L \sum_{\mathbf{k}\sigma} c^\dagger_{S\mathbf{k}\sigma} d_{L\sigma} - t_R \sum_{\mathbf{k}\sigma} c^\dagger_{D\mathbf{k}\sigma} d_{R\sigma} + \text{H.c.}. \tag{9}$$

Here τ_j and t_j (taken to be real) set the strength of the pair (Andreev reflection) and single-electron tunneling between QD and lead j.

As t_L and t_R are the weakest couplings in the system, we call $H_{\text{AMH}} = H_{\text{QD}} + H_{\text{S}} + H_{T,S}$ the Andreev molecular Hamiltonian and treat $H_{T,N}$ as a perturbation to H_{AMH}. That is, the Andreev molecular Hamiltonian gives rise to the Andreev molecular states, and $H_{T,N}$ drives transitions between these states.

Eigenstates of the Andreev molecular Hamiltonian at finite bias

The Andreev molecular Hamiltonian preserves the total electron number N_T, total parity, total spin S_T, and spin projection S_z. Therefore, the Andreev molecular states of the S-QD-QD-S system can be split into subspaces of even and odd parity; the even subspace consists of singlet (S) and triplet ($T_{0,\pm}$) Andreev molecular states, whereas the odd parity subspace consists of doublet ($D_\pm$) Andreev molecular states.

In terms of the number of Cooper pairs in the source and drain leads, N_L and N_R, what do eigenstates of the Andreev molecular Hamiltonian look like at finite bias? A good analogy are the spatially localized eigenstates of a quantum particle in a tilted washboard potential. Although the ground state corresponds to the particle at the "bottom" of the washboard, there is a whole ladder of eigenstates ψ_i, one eigenstate for each lattice site, that

lead to Bloch oscillations. Given the eigenstate ψ_i we can find the state ψ_{i+1} by shifting the wavefunction one lattice site down. Hence the eigenstates can be thought of as forming a ladder, with the rungs labeled by the expectation value for position $\langle x \rangle_i$. Similarly, Andreev molecular states "live" on ladders, with rungs corresponding to the "shift" $s \approx N_L - N_R$ [which will be precisely defined in the next paragraph]. Given an eigenstate on a particular rung, we can obtain the eigenstate on the next rung by shifting a Cooper pair from the left lead to the right lead. For the case of the double quantum dot system there are 16 ladders (4 spin up doublets, 4 spin down doublets, 3 triplets and 5 singlets), which we label by the spin state and "color".

Consider, for example, the singlet Andreev molecular subspace with $N_T = 2N$ electrons. Due to Pauli blockade, there are only five possible ways of electrons occupying the double-dot orbitals, namely $|0,0\rangle, |0,\uparrow\downarrow\rangle, |\uparrow\downarrow,0\rangle, |\uparrow\downarrow,\uparrow\downarrow\rangle$ and $(|\uparrow,\downarrow\rangle - |\downarrow,\uparrow\rangle)/\sqrt{2}$. When coupled to the superconducting leads via Andreev reflection, those five double-dot, singlet states hybridize with the bare states of the superconductors $|N_S, N_D\rangle$, which represent a given distribution of Cooper pairs between the leads. Thus, we can generate any state in this subspace from five reference states of the S-QD-QD-S system, such as $|N,0,0,N\rangle, |N,0,\uparrow\downarrow,N-2\rangle, |N-2,\uparrow\downarrow,0,N\rangle, |N-2,\uparrow\downarrow,\uparrow\downarrow,N-2\rangle$ and $(|N,\uparrow,\downarrow,N-2\rangle - |N,\downarrow,\uparrow,N-2\rangle)/\sqrt{2}$, by transferring Cooper pairs from one lead to the other using the transfer operators $T^{\pm}|N_S, \mathrm{QD_L}, \mathrm{QD_R}, N_D\rangle = |N_1 \pm 2, \mathrm{QD_L}, \mathrm{QD_R}, N_2 \mp 2\rangle$. This is possible since all remaining states of the span correspond to one of the reference states, but with a different Cooper pair configuration. By linearity, the same considerations apply to the eigenstates. Hence, the whole ladder of singlet Andreev molecular states $|S^{(c,s)}\rangle \equiv |N_T = 2N, S_T = 0, S_z = 0, c, s\rangle$ can be constructed from the five reference eigenstates, which we refer to by the "color" quantum number ($c = 1, 2, \ldots, 5$). The number of unique color eigenstates corresponds to the number of unique Andreev molecular states. As a result, the triplet and doublet subspaces can be generated from sets of three and eight color eigenstates, respectively. Here we also introduce the "shift" quantum number ($s = 0, \pm 1, \pm 2, \ldots$), defined as the number of times one needs to apply $T^{\pm}$ to a reference eigenstate to generate an eigenstate with a different Cooper pair configuration.

We define the $s = 0$ reference shift as the eigenstates whose maximum components show minimum Cooper pair imbalance between the leads. We remark that this definition is arbitrary, and alternative definitions should not effect physical results. Note that the

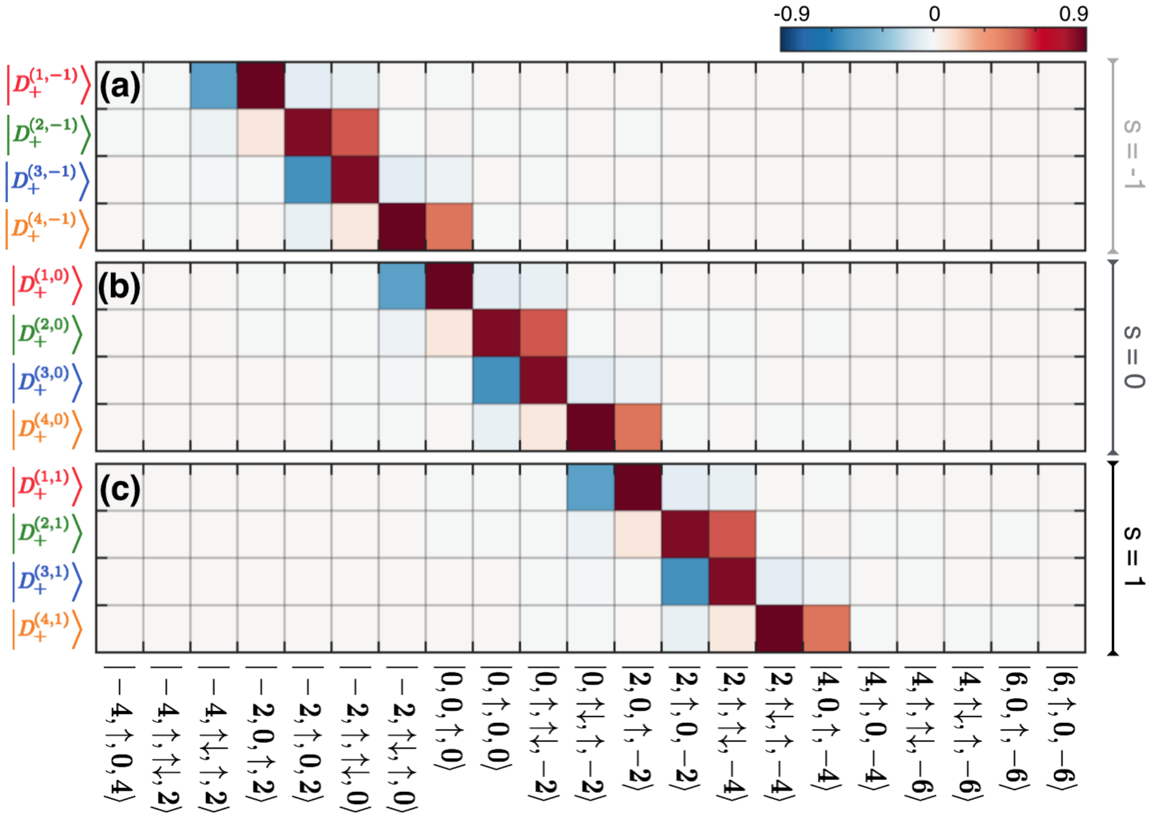

Supplementary Figure 16. Ladder of Andreev molecular states for the doublet subspace $\left|D_+^{(c,s)}\right\rangle \equiv |N_T = 2N+1, S = 1/2, S_z = 1/2, c, s\rangle$ for $V_{\rm bias} = V_D - V_S = 0.2\Delta/e$, $\tau_{L(R)} = 0.8\Delta, t = 0.01\Delta$. We show the set of four color eigenstates corresponding to three shifts (a) $s=-1$, (b) $s=0$, and (c) $s=1$. The $s=0$ reference shift corresponds to the set of color states whose maximum components have minimum Cooper pair imbalance between the leads. From those states, we generate the set of states for the subsequent $s=1$ ($s=-1$) shift by transferring one Cooper pair from the drain (source) lead to the source (drain) lead. For convenience, we choose to count electrons relative to N, which is equivalent to set $N=0$.

eigenenergies for non-zero shifts ($s \neq 0$) can then be easily obtained from the relation

$$E^{c,s}_{N_T,S_z} = E^{c,0}_{N_T,S_z} + 2s\, e(V_S - V_D). \tag{10}$$

As an example, we show in Fig. 16 the $s=0$ color states for the D_+ subspace for different bias voltages. At larger bias voltages, the eigenstates are well localized in Hilbert space, showing a narrow distribution of Cooper pairs. As the bias voltage decreases towards zero, the number of Cooper pairs are allowed to fluctuate and, as a result, the eigenstates spread.

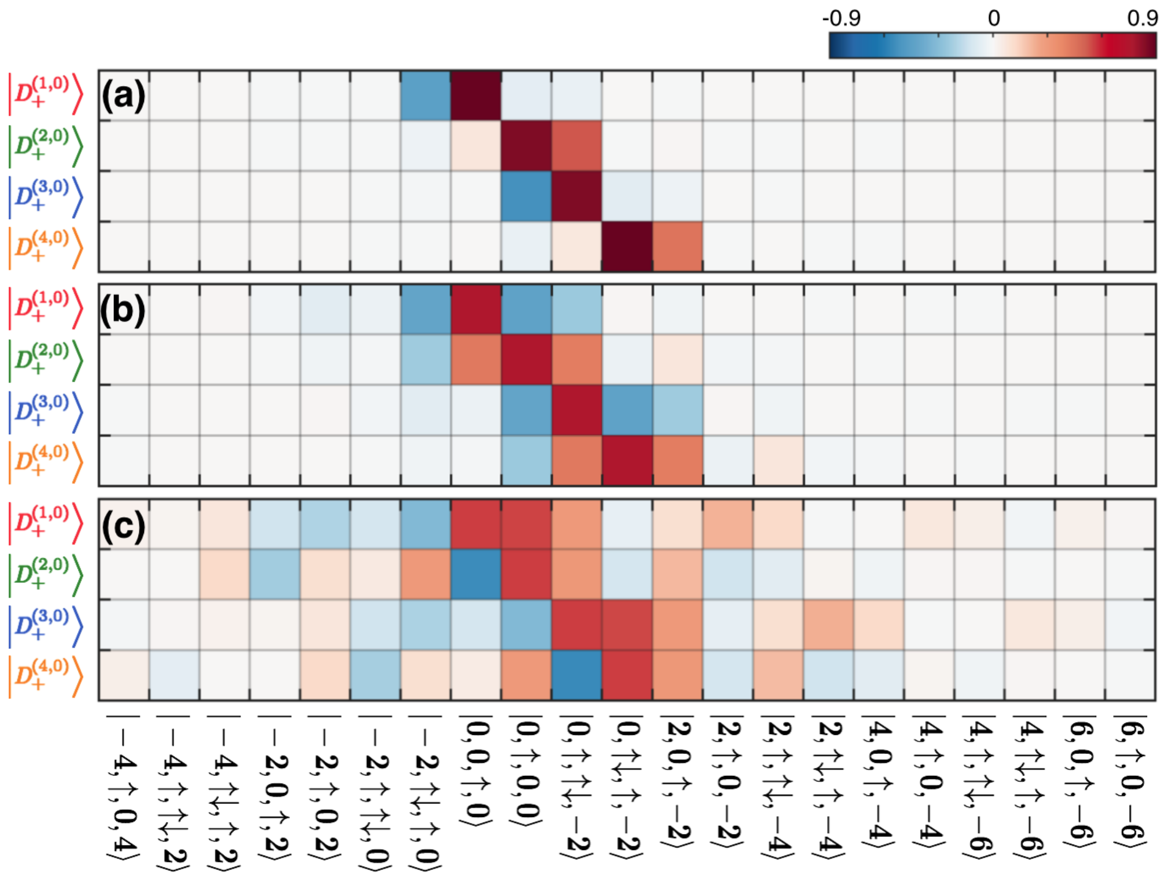

Supplementary Figure 17. Reference $s = 0$ color states for the doublet Andreev molecular subspace $\left|D_+^{(c,0)}\right\rangle \equiv |N_T = 2N+1, S = 1/2, S_z = 1/2, c, s = 0\rangle$ for (a)–(c) $eV_{\text{bias}}/\Delta = 0.2, 0.02, 0.002$, showing the spreading of the probability amplitudes at low bias voltages.

Classical master equation

To describe the experimentally observed sub-gap transport through the S-QD-QD-S device, we now consider the effects of the coupling to the normal component of the leads. We describe the state of the S-QD-QD-S device by the probability distribution P, which gives the probability of finding the system in a particular eigenstate $|N_T, S_T, S_z, c, s\rangle$ of the Andreev molecular Hamiltonian. The S-QD-QD-S system is pushed out of equilibrium by a nonzero source-drain bias voltage. Energy is dissipated by single electrons tunneling from the quantum dots to the normal components of the leads. Such incoherent processes drive transitions between Andreev molecular subspaces of different parity, as illustrated in Fig. 18.

We write a classical master equation that accounts for the transitions between the various

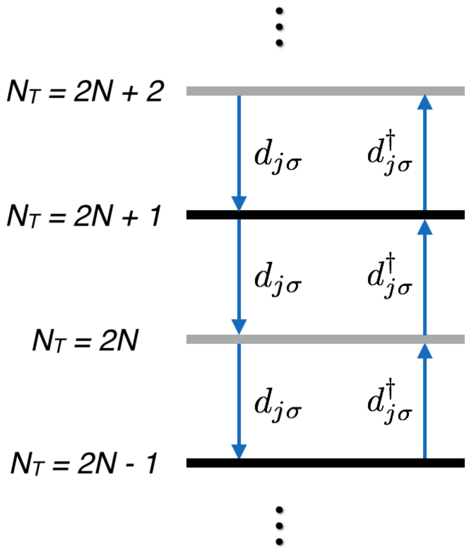

Supplementary Figure 18. Transitions between the even and odd Andreev molecular subspaces of the S-QD-QD-S system driven by single electron tunneling between the normal leads and the double dot subsystem. Depending on the spin of the exchanged electron, these transitions couple doublet states to either singlet or triplet states.

Andreev molecular levels. Depending on the spin of the exchanged electron, these transitions couple doublet states to either singlets or triplets. As we are interested in describing the transport dynamics in the long time limit, the non-equilibrium probability distribution P is given by the steady state solution of the rate equation

$$\frac{\mathrm{d}P(\mathbf{n})}{\mathrm{dt}} = \sum_{\mathbf{m}} \Big(\Gamma_{\mathbf{n}\leftarrow\mathbf{m}} P(\mathbf{m}) - \Gamma_{\mathbf{m}\leftarrow\mathbf{n}} P(\mathbf{n})\Big), \tag{11}$$

where the first (second) term represents the probability of tunneling into (out of) state $|\mathbf{n}\rangle \equiv |N_T, S_T, S_z, c, s\rangle$ and $\Gamma_{\mathbf{n}\leftarrow\mathbf{m}}$ are the transition rates between levels $\mathbf{m}$ and $\mathbf{n}$ due to the exchange of one electron with the normal leads[4]. Specifically, if the transition rate $\Gamma_{\mathbf{n}\leftarrow\mathbf{m}}$ results from the addition of an electron to the S-QD-QD-S system, it is given by

$$\Gamma^{(\mathrm{gain})}_{\mathbf{n}\leftarrow\mathbf{m}} = 2\pi \sum_{j,\sigma} t_j^2 |\left\langle \mathbf{n}\right| d^{\dagger}_{j,\sigma} \left|\mathbf{m}\right\rangle|^2 n_F(E_{\mathbf{n}} - E_{\mathbf{m}} - eV_j), \tag{12}$$

whereas if it results in the loss of an electron to the normal leads, we have

$$\Gamma^{(\mathrm{loss})}_{\mathbf{n}\leftarrow\mathbf{m}} = 2\pi \sum_{j,\sigma} t_j^2 |\left\langle \mathbf{n}\right| d_{j,\sigma} \left|\mathbf{m}\right\rangle|^2 \big(1 - n_F(E_{\mathbf{m}} - E_{\mathbf{n}} - eV_j)\big). \tag{13}$$

Here n_F represents the Fermi-Dirac distribution, which gives the probability to find an electron in the normal leads, and $\{E_{\mathbf{m}}\}$ represent the eigenenergies of the S-QD-QD-S system. The rate equation (11) takes into account all possible single-electron transitions between Andreev molecular states. However, as we show below, we can use the symmetries of the Andreev molecular Hamiltonian to effectively reduce Eq. (11) to involve only transitions between two subspaces of opposite parity, containing a total of $2N$ and $2N+1$ electrons ($N \gg 1$).

As shown in Fig. 18, the $2N$ and $2N+1$ subspaces are directly coupled by transitions involving either the addition of an electron to the $2N$ subspace or the removal of an electron from the $2N+1$ subspace. Using Eqs. (12) and (13), those rates are given by

$$\Gamma^{(\text{gain})}_{\alpha_O \leftarrow \beta_E} = 2\pi \sum_{j,\sigma} t_j^2 |\langle 2N+1, \alpha_O, c', s'| d^\dagger_{j\sigma} |2N, \beta_E, c, s\rangle|^2 n_F\big(E^{c',s'}_{2N+1,\alpha_O} - E^{c,s}_{2N,\beta_E} - eV_j\big), \tag{14}$$

$$\Gamma^{(\text{loss})}_{\alpha_E \leftarrow \beta_O} = 2\pi \sum_{j,\sigma} t_j^2 |\langle 2N, \alpha_E, c', s'| d_{j\sigma} |2N+1, \beta_O, c, s\rangle|^2 \Big(1 - n_F\big(E^{c,s}_{2N+1,\beta_O} - E^{c',s'}_{2N,\alpha_E} - eV_j\big)\Big). \tag{15}$$

For simplicity of notation, here and henceforth we label the spin subspaces by $\alpha_{E(O)}, \beta_{E(O)} = S, T_{0,\pm}(D_\pm)$, where the E, O subscripts emphasize that these are transitions between the $N_T = 2N$ (even) and $N_T = 2N+1$ (odd) Andreev molecular subspaces of the S-QD-QD-S system.

We refer to transitions described by Eqs. (14) and (15) as type 1 transitions. Type 2 transitions connect the $2N$ and $2N+1$ subspaces to the $2N-1$ and $2N+2$ subspaces (see Fig. 18). Type 2 transitions can be mapped back onto the $2N$ and $2N+1$ subspaces because when the number of Cooper pairs is changed by one on a lead at fixed bias voltage, within our model, the eigenenergies are trivially shifted according to the change in electro-chemical potential energy, i.e., $E^{c,s}_{N_T \pm 2,\alpha} = E^{c,s}_{N_T,\alpha} \pm 2eV_j$. Thus, by using this relation and the operators S^+ and S^-, we can write the following identities:

$$\begin{aligned} &|\langle 2N+1, \alpha', c', s'| d_{j\sigma} |2N+2, \alpha, c, s\rangle|^2 \Big(1 - n_F\big(E^{c,s}_{2N+2,\alpha} - E^{c',s'}_{2N+1,\alpha'} - eV_j\big)\Big) \\ &\quad = |\langle 2N+1, \alpha', c', s'| d_{j\sigma} S^+ |2N, \alpha, c, s\rangle|^2 \Big(1 - n_F\big(E^{c,s}_{2N,\alpha} - E^{c',s'}_{2N+1,\alpha'} + eV_j\big)\Big), \end{aligned} \tag{16}$$

$$\begin{aligned} &|\langle 2N+2, \alpha, c', s'| d^\dagger_{j\sigma} |2N+1, \beta, c, s\rangle|^2 n_F\big(E^{c',s'}_{2N+2,\alpha} - E^{c,s}_{2N+1,\beta} - eV_j\big) \\ &\quad = |\langle 2N, \alpha, c', s'| S^- d^\dagger_{j\sigma} |2N+1, \beta, c, s\rangle|^2 n_F\big(E^{c',s'}_{2N,\alpha} - E^{c,s}_{2N+1,\beta} + eV_j\big), \end{aligned} \tag{17}$$

Equations (16) and (17) thus show how transitions of type 2 can be effectively mapped onto a transition between the $2N$ and $2N+1$ subspaces. Note that those transitions are driven by the exchange of a single electron with the normal leads together with the creation or annihilation of a Cooper pair on the superconducting component of the same lead. The rates for type-2 transitions are then given by

$$\tilde{\Gamma}^{(\text{gain})}_{\alpha_E \leftarrow \beta_O} = 2\pi \sum_{j,\sigma} t_j^2 |\langle 2N, \alpha_E, c', s'| S^- d^\dagger_{j\sigma} |2N+1, \beta_O, c, s\rangle|^2 n_F\big(E^{c',s'}_{2N,\alpha_E} - E^{c,s}_{2N+1,\beta_O} + eV_j\big), \tag{18}$$

$$\tilde{\Gamma}^{(\text{loss})}_{\alpha_O \leftarrow \beta_E} = 2\pi \sum_{j,\sigma} t_j^2 |\langle 2N+1, \alpha_O, c', s'| S^+ d_{j\sigma} |2N, \beta_E, c, s\rangle|^2 \Big(1 - n_F\big(E^{c,s}_{2N,\beta_E} - E^{c',s'}_{2N+1,\alpha_O} + eV_j\big)\Big), \tag{19}$$

which describe either the removal of an electron from an even parity state or the addition of an electron to an odd eigenstate.

It is easy to generalize this mapping to all other N_T subspaces and show that any single-electron transition rate are of type 1 or 2 and, hence, can be calculated from Eqs. (14), (15), (18), or (19). Those effective transitions between the $2N$ and $2N+1$ subspaces are illustrated in Fig. 19. In this way, we reduce Eq. (11) to a single even ($2N$) and odd ($2N+1$) subspaces. From now on, we simply refer to those subspaces as even and odd.

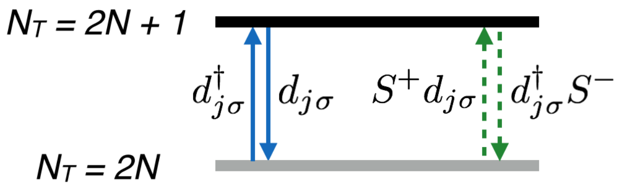

Supplementary Figure 19. Effective transitions between the even ($N_T = 2N$) and odd ($N_T = 2N+1$) Andreev molecular subspaces of the S-QD-QD-S system. The solid, blue arrows represent transitions of type 1, which involve the exchange of an electron between the normal leads and the quantum dots (see Eqs. (14) and (15)). Transitions of type 2 (dashed, green arrows), on the other hand, are driven by the exchange of an electron between the dots and the normal lead, but followed by the creation or annihilation of a Cooper pair in the superconducting component of the same lead (see Eqs. (18) and (19)).

We obtain the steady-state solution of Eq. (11) from the eigenvalue equation

$$\begin{pmatrix} M^{\rm out}_{S} & M^{\rm in}_{S\leftarrow D_+} & M^{\rm in}_{S\leftarrow D_-} & 0 & 0 & 0 \\ M^{\rm in}_{D_+\leftarrow S} & M^{\rm out}_{D_+} & 0 & M^{\rm in}_{D_+\leftarrow T_+} & M^{\rm in}_{D_+\leftarrow T_0} & M^{\rm in}_{D_+\leftarrow T_-} \\ M^{\rm in}_{D_-\leftarrow S} & 0 & M^{\rm out}_{D_-} & M^{\rm in}_{D_-\leftarrow T_+} & M^{\rm in}_{D_-\leftarrow T_0} & M^{\rm in}_{D_-\leftarrow T_-} \\ 0 & M^{\rm in}_{T_+\leftarrow D_+} & M^{\rm in}_{T_+\leftarrow D_-} & M^{\rm out}_{T_+} & 0 & 0 \\ 0 & M^{\rm in}_{T_0\leftarrow D_+} & M^{\rm in}_{T_0\leftarrow D_-} & 0 & M^{\rm out}_{T_0} & 0 \\ 0 & M^{\rm in}_{T_-\leftarrow D_+} & M^{\rm in}_{T_-\leftarrow D_-} & 0 & 0 & M^{\rm out}_{T_-} \end{pmatrix} \begin{pmatrix} \vec{P}_S \\ \vec{P}_{D_+} \\ \vec{P}_{D_-} \\ \vec{P}_{T_+} \\ \vec{P}_{T_0} \\ \vec{P}_{T_-} \end{pmatrix} = 0, \qquad (20)$$

where the matrices $M^{\rm in}_{\alpha\leftarrow\beta} = \Gamma_{\alpha\leftarrow\beta} + \tilde{\Gamma}_{\alpha\leftarrow\beta}$ and $M^{\rm out}_{\alpha} = -\sum_{\beta}\left(\Gamma_{\beta\leftarrow\alpha} + \tilde{\Gamma}_{\beta\leftarrow\alpha}\right)$ describe the influx and outflux of probability of subspace α (with $\alpha, \beta = S, D_{\pm}, T_{0,\pm}$). Note that the vectors $\vec{P}_\alpha$ have dimension $d^\alpha_c d^\alpha_s$, where d^α_c (d^α_s) is the number of color (shift) states in subspace α. Similarly, $M^{\rm out}_\alpha$ and $M^{\rm in}_{\alpha\leftarrow\beta}$ are matrices of dimension equal to $d^\alpha_c d^\alpha_s \times d^\alpha_c d^\alpha_s$ and $d^\alpha_c d^\alpha_s \times d^\beta_c d^\beta_s$.

Steady-state Current

The steady-state current is obtained from the rate at which electrons go through the S-QD-QD-S device and is given by

$$I = -e\sum_{\alpha,\beta}\left(\gamma^{\rm (gain)}_{R,\alpha\leftarrow\beta} - \gamma^{\rm (loss)}_{R,\alpha\leftarrow\beta}\right)\vec{P}_\beta \qquad (21)$$

where the matrices $\gamma^{\rm (gain)}_{R,\alpha\leftarrow\beta}$ and $\gamma^{\rm (loss)}_{R,\alpha\leftarrow\beta}$ (of dimension $d^\alpha_c d^\alpha_s \times d^\beta_c d^\beta_s$) provide the current rates for transitions to the odd subspace, which are given by

$$\gamma^{\rm (gain)}_{R,\alpha_O\leftarrow\beta_E} = 2\pi t_j^2 \left|\left\langle \alpha_O^{(c',s')}\right| d^\dagger_{j\sigma}\left|\beta_E^{(c,s)}\right\rangle\right|^2 n_F\big(E^{c',s'}_{\alpha_O} - E^{c,s}_{\beta_E} - eV_j\big)(2s'-2s-1), \qquad (22)$$

$$\gamma^{\rm (loss)}_{R,\alpha_O\leftarrow\beta_E} = 2\pi t_j^2 \left|\left\langle \alpha_O^{(c',s')}\right| S^+ d_{j\sigma}\left|\beta_E^{(c,s)}\right\rangle\right|^2 \Big(1 - n_F\big(E^{c,s}_{\beta_E} - E^{c',s'}_{\alpha_O} + eV_j\big)\Big)(2s'-2s-1), \qquad (23)$$

and for transitions to the even subspace, which are given by

$$\gamma^{\rm (gain)}_{R,\alpha_E\leftarrow\beta_O} = 2\pi t_j^2 \left|\left\langle \alpha_E^{(c',s')}\right| S^- d^\dagger_{j\sigma}\left|\beta_O^{(c,s)}\right\rangle\right|^2 n_F\big(E^{c',s'}_{\alpha_E} - E^{c,s}_{\beta_O} + eV_j\big)(2s'-2s-1), \qquad (24)$$

$$\gamma^{\rm (loss)}_{R,\alpha_E\leftarrow\beta_O} = 2\pi t_j^2 \left|\left\langle \alpha_E^{(c',s')}\right| d_{j\sigma}\left|\beta_O^{(c,s)}\right\rangle\right|^2 \Big(1 - n_F\big(E^{c,s}_{\beta_O} - E^{c',s'}_{\alpha_E} - eV_j\big)\Big)(2s'-2s-1). \qquad (25)$$

Numerical simulations

We solve the master equation (20) and compute the current (21) numerically, by first finding the set of even and odd eigenstates of the S-QD-QD-S Hamiltonian (4) via exact diagonalization. As discussed above, we restrict this calculation to even and odd subspaces with a total of $2N$ and $2N+1$ electrons. Note that because of the conservation of the total electron number, the exact value of N only sets an overall offset and, hence, N can be taken as an arbitrary parameter. After diagonalizing H, we select the reference color eigenstates and eigenenergies for each subspace. Together with the Cooper pair transfer operators $T^{\pm}$, we then construct the ladders of Andreev molecular states, whose energies are calculated from Eq. (10). We use the ladders of Andreev states to compute the transition probabilities between the even and odd subspaces and their respective Fermi electron (hole) occupation probabilities on the normal leads. Finally, this allow us to calculate the transition rates (14), (15), (18), (19), and (22)–(25) and then to construct and solve both the master equation (20) and the current equation (21).

SUPPLEMENTARY REFERENCES

---

[1] S. Nadj-Perge, V. S. Pribiag, J. W. G. van den Berg, K. Zuo, S. R. Plissard, E. P. A. M. Bakkers, S. M. Frolov, and L. P. Kouwenhoven. Spectroscopy of spin-orbit quantum bits in indium antimonide nanowires. *Phys. Rev. Lett.*, 108:166801, 2012.

[2] Henrik A. Nilsson, Philippe Caroff, Claes Thelander, Marcus Larsson, Jakob B. Wagner, Lars-Erik Wernersson, Lars Samuelson, and H. Q. Xu. Giant, level-dependent g factors in insb nanowire quantum dots. *Nano Letters*, 9(9):3151–3156, 2009.

[3] Sebastian Pfaller, Andrea Donarini, and Milena Grifoni. Subgap features due to quasiparticle tunneling in quantum dots coupled to superconducting leads. *Physical Review B*, 87(15), April 2013.

[4] C. W. J. Beenakker. Theory of coulomb-blockade oscillations in the conductance of a quantum dot. *Phys. Rev. B*, 44:1646–1656, Jul 1991.
